# Self-care guidelines for patients in the post-hematopoietic stem cell transplantation period: a scoping review

**DOI:** 10.1590/0034-7167-2022-0383

**Published:** 2023-10-09

**Authors:** Anália Andréia de Araújo Nascimento, Jéssica Cristina Alves de Melo, Katiane Domingos Soares, Anne Caroline Lisboa Marinho, Sara Eloise Argimiro Ribeiro, Isabelle Campos de Azevedo

**Affiliations:** I Universidade Federal do Rio Grande do Norte. Natal, Rio Grande do Norte, Brazil

**Keywords:** Nursing Care, Patient Discharge Summaries, Hematopoietic Stem Cell Transplantation, Self Care, Health Education, Atención de Enfermería, Resumen del Alta del Paciente, Trasplante de Células Madre Hematopoyéticas, Autocuidado, Educación en Salud, Cuidados de Enfermagem, Sumários de Alta do Paciente Hospitalar, Transplante de Células-Tronco Hematopoéticas, Autocuidado, Educação em Saúde

## Abstract

**Objective:**

To map the evidence on self-care guidelines for patients in the post-hematopoietic stem cell transplantation (HSCT) period.

**Method:**

Scoping review supported by Joanna Briggs Institute recommendations, with searches conducted between March and April 2022 in national and international databases and repositories of theses and dissertations.

**Results:**

Of the 11 studies that composed the final sample, the guidelines had a social and personal aspect, as post-transplant patients need to follow numerous essential recommendations for the prevention of infections and complications for successful treatment and improved quality of life.

**Conclusion:**

Knowing the self-care guidelines that must be performed by post-HSCT patients is fundamental for the nursing team to provide the necessary information for care outside the controlled environment of the hospital, in addition to minimizing episodes of infection, death, and increasing the survival and quality of life of transplant recipients.

## RESUMO

**Objetivo:**

mapear as evidências sobre as orientações realizadas para o autocuidado de pacientes no pós-transplante de células-tronco hematopoéticas (TCTH).

**Método:**

Scoping Review apoiada nas recomendações do Joanna Briggs Institute, com buscas entre março e abril de 2022 em bases de dados e repositórios de teses e dissertações nacionais e internacionais.

**Resultados:**

dos 11 estudos que compuseram a amostra final, as orientações tinham cunho social e pessoal, visto que o paciente do pós-transplante precisa seguir inúmeras recomendações imprescindíveis para a prevenção de infecções e complicações para o êxito do tratamento e melhoria da qualidade de vida.

**Conclusão:**

Conhecer as orientações para o autocuidado que devem ser realizadas por pacientes no pós-TCTH é fundamental para que a equipe de Enfermagem forneça as informações necessárias para os cuidados fora do contexto controlado do ambiente hospitalar, além de minimizar os episódios de infecção, morte e aumentar a sobrevida e qualidade de vida dos transplantados.

## RESUMEN

**Objetivo:**

mapear las evidencias sobre las orientaciones realizadas para el autocuidado de pacientes en el post-trasplante de células madre hematopoyéticas (TCTH).

**Método:**

Scoping Review apoyada en las recomendaciones del Instituto Joanna Briggs, con búsquedas entre marzo y abril de 2022 en bases de datos y repositorios de tesis y disertaciones nacionales e internacionales.

**Resultados:**

de los 11 estudios que compusieron la muestra final, las orientaciones tenían un carácter social y personal, ya que el paciente en el post-trasplante necesita seguir numerosas recomendaciones imprescindibles para la prevención de infecciones y complicaciones para el éxito del tratamiento y la mejora de la calidad de vida.

**Conclusión:**

Conocer las orientaciones para el autocuidado que deben ser realizadas por pacientes en el post-TCTH es fundamental para que el equipo de enfermería proporcione la información necesaria para los cuidados fuera del contexto controlado del ambiente hospitalario, además de minimizar los episodios de infección, muerte y aumentar la sobrevida y calidad de vida de los transplantados.

## INTRODUCTION

Hematopoietic Stem Cell Transplantation (HSCT) is a procedure performed in patients with onco-hematological, immunological, autoimmune, metabolic diseases, and some solid tumors. This treatment is carried out through the transfusion of hematopoietic progenitor cells (HPC), which give rise to new types of cells through the important ability of differentiation and the great capacity of self-renewal^(1-3)^.

Although the HSCT process is considered safe and highly specialized, it is also complex and requires effective care from the multiprofessional team, especially from nursing, which provides 24-hour bedside care to the patient. This procedure involves five stages, namely: conditioning phase, bone marrow infusion, also known as day zero (D0), neutropenic phase, graft or “marrow take,” and finally, post-graft care^(4)^.

The immediate post-transplant period is marked by the first 100 days. It begins one day after HPC infusion, and care can take place in a hospitalization unit if the patient is hospitalized and/or in a Day Hospital and home environment if the procedure occurs on an outpatient basis ^(5)^. Depending on the type of HSCT performed, in the late period, marked by more than 100 days after the procedure, the patient has already definitively returned home or is still receiving outpatient follow-up related to their current clinical condition^(6)^.

This study will focus on the post-HSCT period, which begins immediately after hospital discharge until 100 days after HSCT. It is during this period that the patient becomes primarily responsible for their own care and, as a result, shows more doubts and difficulties in following a new lifestyle that favors the success of the transplant, such as non-adherence to pharmacotherapy due to the numerous medications that they need to take at different times of the day, in addition to the expected and adverse events that some may cause^(7)^.

The success of HSCT is related to the cellular proliferation of the new marrow and the proper functioning of the immune system. However, the failure of HSCT, characterized by graft failure or rejection and disease recurrence, can be related to any stage of the procedure. In the immediate and late periods, the patient may present complications, such as infections, toxicities, graft-versus-host disease (GVHD), and graft failure or rejection, which are the main causes of long hospitalizations and even death^(8-9)^.

Given the above, it is understood the importance of safe care at all stages of HCT, especially in the post-transplant period, when the patient resumes their routine outside the hospital environment and complications may arise that affect various organs and systems, in addition to the increased risk of infections due to the new hematopoietic and immune system still in formation^(10)^.

Nursing guidance for self-care in the post-HCT period can be considered important strategies for a better understanding of the patient about their health-disease process and adherence to treatment. Furthermore, it is important to maintain an open and continuous communication channel with the team for returning to the service whenever necessary^(11-12)^.

Therefore, teaching and encouraging self-care with a view to developing skills and competencies for the early identification of signs and symptoms related to possible complications can minimize undesirable outcomes and strengthen the safety and quality of care with attitudes and practices that can guide the patient to the success of the procedure^(13-14)^.

It is worth noting the importance of knowing and keeping up-to-date on the necessary content for assertive guidance at the time of hospital discharge. Thus, planning educational actions for patient education, with content that should be addressed when preparing for the post-HCT experience, is one of the challenges faced by the nursing team^(15)^.

In this perspective, to achieve the proposed objective, the following research question was elaborated: what content is covered in the guidance for self-care provided to patients in the post-HSCT period?

## OBJECTIVE

To map the evidence regarding guidance provided for self-care of patients in the HSCT period.

## METHODS

### Ethical aspects

There was no need for ethical approval, as the material used is considered public domain as it consists of secondary data. However, it is important to highlight that copyright was respected with the correct citation and referencing.

### Study design

This is a Scoping Review (ScR), guided by the method proposed by the Joanna Briggs Institute (JBI)^(16)^, which followed the recommendations of the Preferred Reporting Items for Systematic Reviews and Meta-Analyses extension for Scoping Reviews (PRISMA-ScR): Checklist and Explanation^(16-17)^. The review protocol was registered on the Open Science Framework (OSF) (https://doi.org/10.17605/OSF.IO/TBRMF).

This method aims to investigate and map the main evidence for a specific area of knowledge through the search of available scientific productions and possible gaps on the topic addressed^(18)^. In order to maintain the reliability of the adopted method, the construct was developed based on five stages: (1) development of the research question; (2) search for relevant studies; (3) selection of studies; (4) extraction and analysis of data; and (5) grouping, summary, and presentation of results^(16)^.

### Methodological procedures

The first stage concerns the elaboration of the research question, for which the PCC strategy was adopted (P = Population: Patients; C = Concept: Hospital patient discharge summaries; and C = Context: Hematopoietic stem cell transplantation). The guiding question determined for the evidence searches was: What content is covered in self-care guidelines for patients after HSCT?

Initially, in order to ensure that there were no studies with the same theme published in the available literature or registered in the OSF, a broad search was conducted on the platform and databases for the identification of protocols or reviews with equivalent themes. Based on this diagnosis, the steps for consolidating the scoping review were followed.

The second stage was subdivided into three phases, the first of which corresponded to the selection of descriptors in published research available in the National Library of Medicine (PubMed) and Cumulative Index to Nursing and Allied Health Literature (CINAHL) databases, according to the JBI manual^(16)^. In the second phase, the defined descriptors were searched in the Medical Subject Heading Terms (MeSH), namely: patients; patient discharge summaries; HSCT; and bone marrow transplantation. The third phase consisted of the database searches, which occurred between March 1 and April 15, 2022.

To capture a larger number of studies related to the object of this research, two distinct search strategies were traced using the Boolean operators AND and OR: patients OR (education, patient OR patient education OR education of patients) AND patient discharge summaries OR (discharge, patient OR discharge planning) AND hematopoietic stem cell transplantation OR (hematopoietic stem cell OR hematopoietic progenitor cells); and patients OR (education, patient OR patient education OR education of patients) AND patient discharge summaries OR (discharge, patient OR discharge planning) AND bone marrow transplantation OR (bone marrow cell transplantation).

### Data sources

The databases listed for the searches were: National Library of Medicine (PubMed); Scopus; Web of Science (WoS); Science Direct, Latin American and Caribbean Health Sciences Literature (LILACS), COCHRANE, The National Library of Australia’s Trove (TROVE), Academic Archive Online (DIVA), Coordination of Improvement of Higher Education Personnel (CAPES), Education Resources Information Center (ERIC), DART-Europe E-Theses Portal, Electronic Theses Online Service (EthOS), Scientific Repository of Open Access of Portugal (RCAAP), National ETD Portal, Theses Canada, Latin American Theses and Dissertations. The adopted strategies underwent some adjustments according to the requirements of each database or repository, as described in Chart 1.

**Chart 1:** Search strategies used in databases and theses and dissertations repositories

Database or Repository	Search strategy used
LILACS, SCIENCE DIRECT, CAPES Theses and Dissertations Catalog, Latin American Theses and Dissertations	#1- *patients* AND *patient discharge summaries* AND *hematopoietic stem cell transplantation* #2- *patients* AND *patient discharge summaries* AND *bone marrow transplantation*
PUBMED, SCOPUS, WoS, COCHRANE, TROVE, DIVA, ERIC, DART-Europe E-Theses Portal, EthOS, RCAAP, National ETD Portal, Theses Canada.	#1- *patients* OR (*education, patient* OR *patient education* OR *education of patients*) AND *patient discharge summaries* OR (*discharge, patient* OR *discharge planning*) AND *hematopoietic stem celltransplantation* OR (*hematopoietic stem cell* OR *hematopoietic progenitor cells*) #2- *patients* OR (*education, patient* OR *patient education* OR *education of patients*) AND *patient discharge sumaries* OR (*discharge, patient* OR *discharge planning)* AND *bone marrow transplantation* OR (*bone marrow cell transplantation*)

### Data Collection and Organization

In the third phase of the second stage, according to JBI recommendations^(16)^, the studies retrieved from repositories were screened by four reviewers who worked in pairs to select potential eligible studies by reading their titles and abstracts. There was no disagreement regarding the inclusion of the selected materials, and only studies that met the research objective, addressed the guiding question, and were available in full text through remote access to the Federated Academic Community (CAFe) via the CAPES Ministry of Education Periodicals Portal were included. Studies in editorial, letter to the editor, and opinion article formats were excluded. It should be noted that duplicate studies were considered only once. According to JBI recommendations^(16)^, no time frame was applied, since the aim of this type of review is to map all possible studies on the topic.

In the third stage, the four researchers independently selected the pre-analyzed studies by applying eligibility criteria. The fourth stage involved an expert in the field reading the studies in full to extract data. These stages took place between April 16 and 30, 2022.

The article titles and authors were used to differentiate them during the data extraction process, which was recorded in a Microsoft Office® 2019 Excel spreadsheet, collecting the following variables: language, publication year, country where the study was conducted, methodological design, guidance provided to patients after HSCT, and level of evidence classified according to JBI^(19)^.

### Data analysis

**Figure 1:**
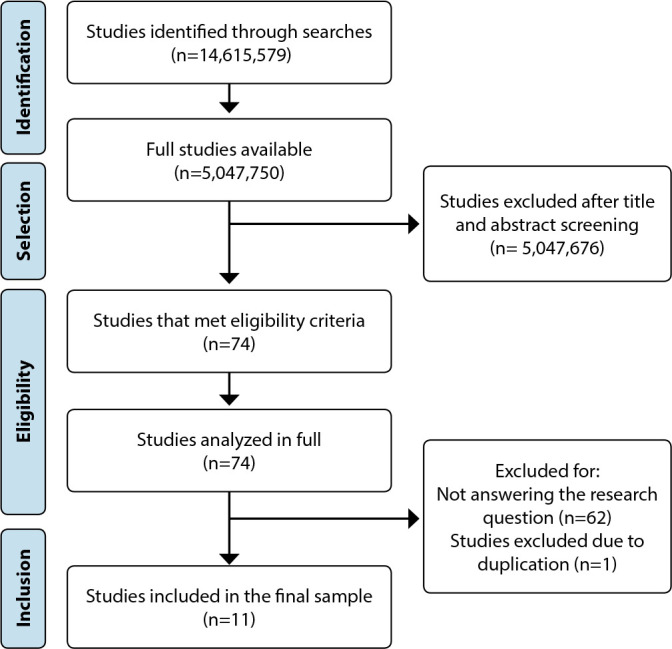
Flowchart of the selection process, 2022 (N=11)

The collected data were organized and analyzed descriptively by presenting relative and absolute frequencies. The search results are displayed in Figure 1, and the analyzed data are presented in paragraphs in the Results section and in Table 1. They were discussed with support from the literature on the subject, which represents the fifth stage of the adopted method. To differentiate the studies, they were assigned an alphabet letter followed by an Arabic numeral to identify them, for example: A1, A2, A3, etc. It should be noted that the list of studies that comprised the final sample, as well as their respective references, is attached to the OSF platform.

## RESULTS

Initially, 14,615,579 studies were identified in the listed databases and thesis and dissertation repositories, but only 5,047,750 were available in complete format for analysis. After the selection process described in Figure 1, 11 articles composed the final sample.

Regarding the year, it was found that in 2021, four articles (36.37%) addressed the guidelines provided to patients after HSCT, followed by 2020 with only two (18.18%). In the years 2019, 2018, 2016, 2014, and 2011, one study (9.09%) was published per year. With regard to language, all studies (11; 90.91%) are in English.

Among the countries where the research was conducted, Brazil (3; 27.28%) stands out, followed by the United States of America (USA) and Turkey, with two articles (18.18%) each, and Germany, Canada, Italy, and France with one publication (9.09%) per country.

Chart 2 shows the detailed characterization of the selected studies, regarding reference, year and country of publication, type of study, summary of self-care guidelines provided to patients after HSCT, and level of evidence.

**Chart 2:** Characterization of studies included in the scoping review, 2022 (N=11)

Author / Title	Year / Country	Design / Number of participants^*^	Self-care guidance provided to patient’s post-HSCT	Evidence level
Nunes et al.^(11)^/Adherence to nursing guidelines in relation to home care of bone marrow transplantees in the ecosystem perscpective	2020 / Brazil	Qualitative / n=40	Care with pets; Use of cosmetics and skin care; Avoid direct contact with infected individuals and/or groups of people; Care with plants; Guidance on contact with sharp materials; Household hygiene; Care for sexual relationships; Guidance on caring for family health.	IV^†^
Akbulut et al.^(20)^/Overview of nutritional approach in hematopoietic stem cell transplantation: COVID-19^‡^ update	2021 / Turkey	Review	Nutritional guidance; Food hygiene	IV^†^
Bertz ^(21)^/Rehabilitation after Allogeneic Haematopoietic Stem Cell Transplantation: A Special Challenge	2021/ Germany	Review	Self-care; Nutritional guidance; Food hygiene; Personal hygiene; Household hygiene; Medications; Avoiding direct contact with infected individuals and/or groups; Vaccination; Care with plants; Care with domestic animals; Psychological support; Returning to work; Ambulatory appointments; Laboratory exam control.	IV^†^
Barban et al.^(22)^/Analysis of the feasibility of early hospital discharge after autologous hematopoietic stem cell transplantation and the implications to nursing care	2014 / Brazil	Cross-sectional / n=100	Adverse effects of treatment; Recognition of signs and symptoms of possible complications; Seeking professional help; Hydration intake; Catheter care; Laboratory test control.	III.2^§^
Polito et al.^(23)^/Evaluation of a patient self-medication program in allogeneic hematopoietic stem cell transplantation	2021 / Canada	Cohort / n=51	Medications.	III.2^§^
Oguz et al.^(24)^/Sintomas após alta hospitalar após transplante de células-tronco hematopoiéticas	2016 / Turkey	Descriptive / n=66	Orientations on management and common symptoms in the post-HSCT period.	IV^†^
Song et al.^(25)^/It Takes a Team to Make It Through: The Role of Social Support for Survival and Self-Care After Allogeneic Hematopoietic Stem Cell Transplant	2021/ USA^||^	Qualitative / n=173	Nutritional guidelines; Medications; Psychological support.	IV^†^
Faucher et al.^(26)^/Randomized study of early hospital discharge following autologous blood SCT^¶^: medical outcomes and hospital costs	2011 / France	Randomized Clinical Trial / n=131	Monitoring body temperature; Medication; Need for staying/lodging near the referral hospital.	II^^**^^
Denzen et al.^(27)^/Tailoring a Survivorship Care Plan: Patient and Provider Preferences for Recipients of Hematopoietic Cell Transplantation	2019 / USA^||^	Qualitative/n=77	Recognition of signs and symptoms of possible complications; Toxicity; Vaccination.	IV^†^
Cioce et al.^(28)^/Impact of educational interventions on psychological distress during allogeneic hematopoietic stem cell transplantation: a randomised study	2020 / Italy	Randomized Clinical Trial / n=36	Recognition of signs and symptoms of possible complications; Self-care; Nutritional guidance; Food hygiene; Adverse effects of treatment; Attention to medication side effects; Risks and preventive behavior; Hand hygiene; Protective isolation.	II^^**^^
Rodrigues et al.^(29)^/Research contributions for the Nursing care in pediatric transplantation of hematopoietic stem cells	2018 / Brazil	Qualitative / n=138	Nutritional guidance; Food hygiene; Use of masks; Protective isolation; Hand hygiene; Vital signs monitoring; Laboratory test control.	IV^†^

Note:

* The number of participants was included in studies involving human subjects; †Evidence from expert opinions, narrative literature reviews, and descriptive studies, according to the Joanna Briggs Institute Manual^(16)^; ‡Coronavirus Disease 2019; §Evidence obtained from well-designed cohort or case-control studies, analytical studies, according to the Joanna Briggs Institute Manual^(16)^; ||United States of America; ¶Stem Cells Transplantation;

** Evidence obtained from at least one randomized controlled clinical trial, according to the Joanna Briggs Institute Manual^(16)^.

## DISCUSSION

From the analyzed studies, several guidelines emerged that are necessary for teaching and maintaining self-care based on the new lifestyle habits that the transplant recipient and their caregivers/family members must follow to enable quality of life, prevent infections, and complications after hospital discharge. Thus, the listed guidelines are not only directed at caring for the patients’ health and illness processes in the immediate post-HSCT period, when the patient is discharged, but also in relation to the environment surrounding them^(11)^.

The contents covered in the selected studies for the final sample refer to the domestic/family/social ecosystem and are related to precautions, such as avoiding contact with people with fever, cough, flu, among others^(11)^, as well as changes in home organization and hygiene^(11,22)^, since transplant recipients have an immature immune system with a high risk for opportunistic infections or other diseases and, for this reason, must comply with several restrictions throughout the post-HSCT period^(23)^.

Respiratory viral infections are some of the causes of morbidity and mortality in the post-HSCT period and can be minimized through the use of masks, hand hygiene, avoiding handshakes, and environments where contaminated people are present^(30)^. Regarding home care, this requires a separate room and bathroom for the transplant recipient, restriction of the number of visitors, and a caregiver to clean the environment^(12)^.

Regarding the implementation of new lifestyle habits, self-care^(20,22)^, nutritional guidelines^(20-21,25,28-29)^, food hygiene^(20-21,25, 28)^, fluid intake^(20)^, hand^(25,28)^ and personal hygiene^(21)^, correct medication use^(20-21,23,26)^, and complications^(22,27-28)^ were the most cited in the studied sample.

Patients should be encouraged to practice self-care and self-management of their health condition even during hospitalization, so they do not have difficulty following the instructions given. Regarding HSCT, this treatment requires that patients and their families/caregivers are capable of collaborating for effective care, following the care guidelines, to minimize risks and have a better quality of life^(31)^, from self-care to the correct management of complications.

Nutritional care after hospital discharge refers to precautions regarding food hygiene and preparation, as well as the ingestion of raw or contaminated foods (meat, milk, vegetables, fruits, grains, bread, pastries, unpackaged cakes, cream cakes, dried fruits, nuts, or coconut), in addition to untreated water. Therefore, it is important to handle, prepare, and consume food properly to avoid infections transmitted by microorganisms. To do so, the following precautions must be taken: cleaning - wash hands, surfaces, and products with soap and water; separation - to avoid cross-contamination, store food and water properly; cooking - cook food and water at the appropriate temperature; and refrigeration - refrigerate immediately^(20)^.

Water consumption is another important aspect to be discussed, as patients present renal overload due to the toxicity related to chemotherapy, which persists for a few months post-HSCT, and for this reason, they require adequate oral hydration. Aspects such as treatment, commercialization, and distribution of water must be considered. Water is considered treated when filtered or boiled for at least 2 minutes, or filtered and chlorinated. In cities where there is no water supply network, the addition of one drop of sodium hypochlorite per liter of consumed water is recommended^(2)^.

Another guidance that requires constant attention from patients/families/caregivers is hand hygiene. This practice is recommended as the most effective isolated measure for the prevention and control of infections and is also considered one of the pillars of patient safety, according to the World Health Organization (WHO)^(32-33)^.

In terms of hygiene, maintaining proper cleanliness is essential in reducing the risk of opportunistic diseases caused by microorganisms that naturally inhabit the human microbiota. It is recommended to take a daily shower with a neutral soap, paying particular attention to washing the armpits and groin. It is also important to use a personal towel, changing it frequently, hydrate the skin, limit exposure to sunlight, and use sunscreen daily. If contact lenses are used, they should be worn for a limited period. Makeup products can be used, but only if they are new and intended for individual use. It is advisable to postpone visits to the manicurist until the immune system has recovered. If there is an implanted central venous catheter, it should be kept dry, and the dressing should be changed periodically. Piercings and tattoos should be avoided^(12)^.

Following nursing guidance, especially related to medications, is one of the biggest challenges, and this fact correlates with non-adherence to pharmacotherapy, the deficit of social support, and the need for instrumental support from family members/caregivers, who are often responsible for reminding transplant patients of medications and their schedules^(20)^. Furthermore, the effectiveness of communication between the health team and the patient, as well as the quality of this relationship, are also seen as factors linked to patient adherence to treatment, especially to medications^(25)^.

Without proper daily dosing of medications, such as immunosuppressants and others, patients may experience complications that can lead to graft failure, disease relapse, or GVHD. A study conducted in Canada reports on the patient experience with medications at the time of discharge and suggests that patients who are taught and held responsible for their medication treatment while still in the hospital, with the support and supervision of nursing staff, have better outcomes related to self-efficacy and treatment knowledge, as well as a reduction in adverse events or readmissions post-discharge^(23)^.

Regarding the early recognition of possible complications, healthcare professionals show concern about instructing on acute GVHD^(26,28)^. In addition to signs of complications, such as skin changes and gastrointestinal problems^(22)^, the side effects of medications^(29)^, and the expected post-HCT symptoms^(24)^ are taught, which can affect the quality of life of patients. The importance of these topics emerges due to the need for early detection and classification of complications, as well as rapid seeking of professional help^(22)^, consistency in outpatient visits^(21)^, and attention to staying/lodging near the reference hospital^(26)^.

The sample also suggests the importance of routine laboratory tests^(21-22,25)^, especially a complete blood count, as well as monitoring vital signs^(29)^, especially body temperature^(25)^, and care of the central catheter^(13)^, in order to detect infections early and act immediately with antibiotic therapy^(29)^. Fear of infections is natural during this process and is related to the immaturity of the new bone marrow and developing immune system^(34)^. Therefore, instructions need to be started as soon as possible so that the patient understands and applies them upon returning home. Such instructions include: avoiding caring for plants, domestic animals, and using sharp objects; using prescribed cosmetics, especially sunscreen; and using a condom during sexual intercourse^(11)^.

Another guidance cited as important for continuing treatment in late post-transplant is the need to restart practically the entire vaccination scheme^(21,27)^ due to the loss of a large part of the defense cells destroyed by the disease and chemotherapy. The protocol suggested by the Ministry of Health for vaccination of transplant patients with HCT considers the start time of the scheme from three to 12 months after HCT, depending on the clinic and recommendations of the medical team^(35)^. The guidelines of the 2017 European Conference on Infections in Leukemia (ECIL 7) indicate that immunobiologics should be administered from six months after D0^(36)^. Even after administration of the vaccines, the patient’s transplanted immune system is still considered fragile, which justifies reinforcing nursing instructions regarding timing and return for subsequent doses and care when returning to work^(21)^ and contact with large groups that tend to have infected people^(11,20)^. Therefore, it is necessary for the healthcare professional to ensure that the patient understands the importance of protective isolation^(28-29)^ and mask use, so that the patient has awareness and tendency to avoid risky behaviors^(28)^. Returning home with so many restrictions can lead to mental health problems. Therefore, it is valid to emphasize the need for psychological support^(21,25)^. A study of 272 patients showed that HCT, whether autologous or allogeneic, is a treatment that triggers psychological disorders, such as anxiety and depression, in any of its phases, from pre to post-procedure^(37)^.

Related to the willingness to learn the necessary guidelines for the continuation of treatment, a study conducted a focus group with patients and caregivers post-HSCT and showed their desire to have didactic and easily accessible materials containing discharge instructions, particularly a section dedicated to topics such as sexual and reproductive health, emotional well-being, cancer, immune system, and guidance on resuming daily activities, in order to feel more secure about these issues^(27)^. However, research with patients in Brazil and Spain shows that the amount of information provided in discharge instructions left transplant patients with doubts and uncertainties about the care that should be provided at home. Other participants make it clear in their speeches that they do not follow important guidelines, such as using sunscreen, condoms for sexual intercourse, and taking care of their diet^(11)^.

It is understood that the guidelines contained in the sample are a reflection of the necessary information for the conduct of a better quality of life and safety in decision-making by patients in the post-HSCT period. The collected data allowed the interpretation that often patients do not perform self-care guidelines because they do not understand their relevance to the treatment follow-up. Perhaps providing feedback and/or asking the patient to repeat the information provided after the guidelines can decrease doubts and difficulties.

Furthermore, considering the numerous subjects addressed at once, it is considered essential to use accessible tools and/or technologies with content that reaches different levels of education and socioeconomic status to support nursing guidelines in preparing patients to experience the post-HSCT period.

### Limitations of the Study

This study presents limitations regarding the predominance of studies with low levels of evidence, as the methodologies come from reviews and qualitative studies. The present review focused on self-care guidelines provided to patients after HSCT, which may have decreased the sample population. Despite these limitations, this review stands out for the methodological rigor required by JBI and the mapping of the main evidence on the topic.

### Contributions to Nursing, Health, or Public Policy

The contributions to the health and nursing field are based on the impact that health education has on the management of self-care for chronic patients. Therefore, studies like this, which explore the need to properly guide patients to assume self-care in new experiences and lifestyles after HCT, as well as offer materials with academic rigor and easy access with didactic content that meet the knowledge and professional development needs of teams working with such specific patients, have significant scientific value.

## CONCLUSION

It is concluded that for patients experiencing the immediate post-HSCT period, correct and effective nursing self-care instructions are crucial for the continuity and success of their treatment. Home self-care instructions are inherent to the nursing team’s work and should be based on the best current scientific evidence.

Understanding the self-care instructions that must be performed by patients during this period is fundamental for the healthcare team, especially nursing, to provide the necessary information for the self-management of care outside the controlled context of the hospital environment. Thus, this review compiled the main instructions based on studies that discussed the research subject. Such instructions can minimize episodes of infection, other complications, and even death, as well as increase the survival and quality of life of transplant recipients.

Finally, it is highlighted the scarcity of studies in the literature that comprehensively address the necessary content for self-care instructions in the post-transplant period, which emerges as a suggestion for further research in the area. Researchers are also encouraged to conduct experimental studies to demonstrate the efficacy of these instructions developed in research and their impact on the patient’s quality of life in the post-HSCT period.
